# Development and internal validation of a multidimensional nomogram integrating PIV, LDH, and FeNO for predicting poor asthma control in school-aged children

**DOI:** 10.3389/fped.2026.1855307

**Published:** 2026-06-29

**Authors:** Zhijian Zhan, Tianfu Xu, Saiping Huang

**Affiliations:** Department of Paediatrics, The Affiliated Hospital of Putian University, Putian City, Fujian, China

**Keywords:** asthma control, FeNO, lactate dehydrogenase, nomogram, pan-Immune-Inflammation value, pediatric asthma, risk prediction

## Abstract

**Background:**

Poor asthma control remains common in school-aged children despite guideline-based treatment. Traditional assessment tools have limitations, highlighting the need for objective and multidimensional biomarkers. This study aimed to develop and internally validate a risk stratification model integrating systemic inflammatory, metabolic, and airway-specific indicators for identifying uncontrolled asthma.

**Methods:**

In this retrospective study, 232 children with bronchial asthma (aged 6–14 years) were enrolled. Asthma control was assessed using the Childhood Asthma Control Test (C-ACT). Clinical data, laboratory biomarkers, and pulmonary function parameters were collected. Least absolute shrinkage and selection operator (LASSO) regression was used for variable selection, followed by multivariate logistic regression to identify independent predictors. A nomogram was constructed, and internal model performance was evaluated using receiver operating characteristic (ROC) analysis, calibration curves, and decision curve analysis (DCA).

**Results:**

Six variables were identified as independent predictors of poor asthma control: PIV, LDH, FeNO, Vitamin D, asthma duration, and FEV1% predicted. PIV (OR=1.008), LDH (OR=1.043), FeNO (OR=1.056), and asthma duration (OR=1.251) were risk factors, whereas Vitamin D (OR=0.891) and FEV1% predicted (OR=0.953) were protective. The combined model demonstrated superior predictive performance (AUC = 0.886, 95% CI: 0.835–0.937) compared with individual biomarkers and the baseline model. The nomogram showed good calibration and provided favorable clinical net benefit in DCA.

**Conclusions:**

A multidimensional model integrating PIV, LDH, FeNO, Vitamin D, asthma duration, and lung function provides accurate and clinically applicable risk stratification of poor asthma control in school-aged children. This approach may facilitate localized risk assessment and support personalized management in pediatric asthma.

## 1 Introduction

Bronchial asthma is the most common chronic respiratory disease among school-aged children worldwide, imposing a substantial burden on healthcare systems and significantly impairing quality of life (1, 2). It is characterized by chronic airway inflammation, reversible airflow limitation, and airway hyperresponsiveness, with considerable heterogeneity in both clinical presentation and underlying inflammatory mechanisms (3, 4). Despite the widespread implementation of guideline-based therapies, particularly inhaled corticosteroids (ICS), a substantial proportion of pediatric patients continue to experience poor asthma control, leading to recurrent exacerbations, increased healthcare utilization, and long-term airway remodeling (5, 6). Therefore, early identification of children at high risk of uncontrolled asthma remains a critical priority in pediatric respiratory care.

Traditional tools for assessing asthma control, including symptom-based questionnaires such as the Childhood Asthma Control Test (C-ACT) and objective measurements like spirometry, have inherent limitations (7, 8). Symptom-based assessments are susceptible to recall bias and caregiver interpretation, while pulmonary function testing requires a level of cooperation that is often difficult to achieve in younger children (9). These limitations highlight the need for objective, reliable, and non-invasive biomarkers that can better reflect the underlying pathophysiology of asthma and improve risk stratification in clinical practice. Although current guidelines, including those from the Global Initiative for Asthma (GINA), emphasize the importance of biomarker-based assessment, clinically applicable multidimensional biomarkers remain insufficiently explored, particularly in pediatric populations (10).

Increasing evidence suggests that asthma is not solely a localized airway disease but also involves systemic immune-inflammatory dysregulation (11–13). In this context, composite inflammatory indices derived from routine blood parameters have gained growing attention. The Pan-Immune-Inflammation Value (PIV), which integrates neutrophil, lymphocyte, monocyte, and platelet counts, has emerged as a novel and comprehensive marker of systemic inflammation (14, 15). Compared with conventional indices such as the neutrophil-to-lymphocyte ratio (NLR) or systemic immune-inflammation index (SII), PIV may better capture the complex interactions between innate and adaptive immune responses (16, 17). Recent studies have demonstrated its prognostic value in various inflammatory and immune-mediated diseases; however, its role in pediatric asthma, particularly in predicting disease control status, remains largely unexplored.

In addition to immune-inflammatory mechanisms, metabolic dysregulation has been increasingly recognized as a key contributor to asthma pathophysiology (18, 19). Lactate dehydrogenase (LDH), a critical enzyme in anaerobic glycolysis, reflects cellular injury, tissue hypoxia, and inflammatory activation (20, 21). Elevated LDH levels may indicate airway epithelial damage and metabolic reprogramming under inflammatory conditions, thereby providing insight into disease severity and progression (22, 23). Moreover, Vitamin D, an important immunomodulatory factor, has been implicated in the regulation of both innate and adaptive immune responses (24). Vitamin D deficiency has been associated with increased airway hyperresponsiveness, enhanced inflammatory responses, and reduced responsiveness to corticosteroid therapy, further complicating asthma control in children (25, 26).

At the airway level, fractional exhaled nitric oxide (FeNO) serves as a well-established, non-invasive biomarker of type 2 (T2) eosinophilic inflammation (27). FeNO measurement is simple, reproducible, and widely applicable in pediatric populations. Elevated FeNO levels are closely associated with eosinophilic airway inflammation and have been linked to exacerbation risk and therapeutic response (28, 29). However, the predictive performance of FeNO alone for overall asthma control remains inconsistent, likely due to the heterogeneity of asthma phenotypes and the influence of systemic and environmental factors (30). This underscores the limitation of relying on a single biomarker to capture the complexity of asthma.

Given the multifactorial nature of asthma, an integrated approach combining systemic inflammatory markers, metabolic indicators, and airway-specific biomarkers may provide a more comprehensive assessment of disease activity and control status. Machine learning techniques, such as least absolute shrinkage and selection operator (LASSO) regression, offer a robust method for variable selection in high-dimensional datasets, minimizing overfitting and improving model performance. By incorporating these approaches, predictive models can achieve greater accuracy and clinical applicability. Therefore, the present study aimed to integrate systemic (PIV), metabolic (LDH, Vitamin D), and airway (FeNO) biomarkers, along with key clinical variables, to develop and internally validate a contemporaneous risk stratification model for poor asthma control in school-aged children. Given the retrospective nature of this study, the developed model is specifically designed to reflect the real-time disease status and multi-systemic biological burden of patients at evaluation, rather than predicting long-term prospective outcomes independent of current clinical severity. By constructing a nomogram based on multivariate logistic regression, we sought to provide a practical, non-invasive, and clinically applicable tool for early risk stratification and personalized management in pediatric asthma.

## 2 Material

### 2.1 Study design and population

This retrospective, observational study was conducted at the Affiliated Hospital of Putian University between January 2024 and December 2025. The study protocol was reviewed and approved by the Ethics Committee of the Affiliated Hospital of Putian University (Approval No. 2026105). Due to the retrospective nature of the study, which utilized de-identified clinical data and posed no more than minimal risk to the participants, the requirement for written informed consent was formally waived by the Ethics Committee. All procedures were performed in accordance with the ethical standards of the 1964 Declaration of Helsinki and its later amendments. Notably, because clinical biomarkers and scores were collected cross-sectionally during routine clinical follow-ups, the analyzed variables reflect the contemporaneous clinical and biological status of the patients at the time of evaluation.

A total of 232 school-aged children (aged 6–14 years) diagnosed with bronchial asthma were consecutively enrolled. All participants had received standardized maintenance therapy, primarily inhaled corticosteroids (ICS), for at least three months prior to enrollment in accordance with the Global Initiative for Asthma (GINA) guidelines.

Inclusion criteria were as follows: (1) a confirmed diagnosis of pediatric bronchial asthma; (2) age between 6 and 14 years with adequate cooperation for pulmonary function testing; and (3) stable clinical condition without acute exacerbations within the preceding 4 weeks. Exclusion criteria included: (1) the presence of other chronic respiratory diseases such as bronchopulmonary dysplasia or bronchiectasis; (2) concurrent systemic infections, autoimmune diseases, or malignancies; (3) use of systemic corticosteroids or immunosuppressive agents within the past 4 weeks; and (4) incomplete clinical or laboratory data.

### 2.2 Assessment of asthma control

Asthma control status was evaluated using the Childhood Asthma Control Test (C-ACT), a validated questionnaire widely used in pediatric populations. According to GINA recommendations and C-ACT scoring criteria, participants were categorized into two groups: the controlled group (C-ACT score ≥ 20) and the uncontrolled group (C-ACT score < 20).

### 2.3 Data collection and laboratory measurements

Clinical and demographic data, including age, sex, BMI-Z score, perinatal history, and environmental exposures (such as secondhand smoke exposure and pet ownership), were retrieved from electronic medical records.

Peripheral venous blood samples were collected in the morning following an overnight fast. Routine hematological parameters, including neutrophil (N), lymphocyte (L), monocyte (M), and platelet (P) counts, were measured using an automated hematology analyzer (Sysmex XN-9000, Japan). The Pan-Immune-Inflammation Value (PIV) was calculated as PIV=(N*M*P)/L. Additional inflammatory indices, including the systemic immune-inflammation index (SII = P*N/L) and neutrophil-to-lymphocyte ratio (NLR = N/L), were also derived.

Serum biochemical parameters, including lactate dehydrogenase (LDH), Vitamin D, and total immunoglobulin E (IgE), were measured using standard laboratory assays according to manufacturer protocols.

### 2.4 Airway inflammation and pulmonary function

Fractional exhaled nitric oxide (FeNO) was measured using an electrochemical analyzer (NIOX VERO, Sweden) in accordance with the American Thoracic Society/European Respiratory Society (ATS/ERS) guidelines. Pulmonary function tests were performed using a calibrated spirometer (PowerCube, Ganshorn, Germany). The measured parameters included forced expiratory volume in one second (FEV1% predicted), FEV1/forced vital capacity (FEV1/FVC), and small airway function indices, including maximal mid-expiratory flow (MMEF% predicted), forced expiratory flow at 50% of FVC (FEF50% predicted), and forced expiratory flow at 75% of FVC (FEF75% predicted).

### 2.5 Statistical analysis

All statistical analyses were conducted using R software (version 4.3.0) and SPSS (version 26.0). Continuous variables were expressed as mean ± standard deviation (SD) or median with interquartile range (IQR), depending on data distribution, and were compared using the Student's t-test or Mann–Whitney U test as appropriate. Categorical variables were presented as frequencies (percentages) and compared using the Chi-square test or Fisher's exact test.

To identify the most informative predictors and reduce the risk of overfitting, the least absolute shrinkage and selection operator (LASSO) regression model was applied for variable selection. The optimal penalty parameter (λ) was determined through 10-fold cross-validation, and variables with non-zero coefficients were subsequently included in a multivariate logistic regression analysis to identify independent risk factors for poor asthma control.

Based on the final multivariate model, a nomogram was constructed to provide an individualized risk prediction tool. Model performance was evaluated in three aspects: discrimination, calibration, and clinical utility. Discrimination was assessed using the area under the receiver operating characteristic curve (AUC), and the DeLong test was employed to evaluate the statistical significance of differences in AUCs between the combined model, individual biomarkers, and the clinical baseline model. Calibration was evaluated using calibration plots and the Hosmer–Lemeshow goodness-of-fit test. Clinical utility was assessed using decision curve analysis (DCA), which quantified the net benefit across a range of threshold probabilities. To ensure robust internal validation and account for potential overfitting, both the calibration curves and decision curve analysis were evaluated using a bootstrap resampling technique with 1,000 replicates.

A two-sided *P*-value < 0.05 was considered statistically significant.

## 3 Results

### 3.1 Baseline demographic and clinical characteristics of the study population

A total of 232 school-aged children with bronchial asthma were included in this study, comprising 128 patients in the controlled group and 104 patients in the uncontrolled group. As shown in Table 1, there were no significant differences between the two groups in age, sex distribution, BMI-Z score, cesarean delivery, preterm birth, low birth weight, or pet ownership (all *P* > 0.05). However, exposure to secondhand smoke was significantly more frequent in the uncontrolled group than in the controlled group (45.2% vs. 28.1%, *P* = 0.01).

**Table 1:** Demographic and clinical characteristics of the study population.

Variables	Controlled (*n* = 128)	Uncontrolled (*n* = 104)	*P*-value
Demographic characteristics			
Age, years	9.45 ± 2.10	9.28 ± 2.25	0.553
Male sex, *n* (%)	83 (64.8%)	65 (62.5%)	0.816
BMI-Z score	0.25 ± 0.88	0.38 ± 0.95	0.281
Perinatal & environmental factors			
Cesarean delivery, *n* (%)	54 (42.2%)	47 (45.2%)	0.744
Preterm birth, *n* (%)	15 (11.7%)	16 (15.4%)	0.534
Low birth weight, *n* (%)	10 (7.8%)	11 (10.6%)	0.617
Secondhand smoke exposure, *n* (%)	36 (28.1%)	47 (45.2%)	0.01
Pet ownership, *n* (%)	23 (18.0%)	22 (21.2%)	0.658
Clinical history			
Age at onset, years	4.12 ± 1.85	3.75 ± 2.10	0.155
Asthma duration, years	4.55 ± 2.10	5.85 ± 2.65	<0.001
Allergic rhinitis, *n* (%)	74 (57.8%)	78 (75.0%)	0.009
Eczema, *n* (%)	41 (32.0%)	46 (44.2%)	0.076
Food allergy, *n* (%)	15 (11.7%)	23 (22.1%)	0.037
Family history, *n* (%)	45 (35.2%)	54 (51.9%)	0.015

With regard to clinical history, asthma duration was significantly longer in the uncontrolled group compared with the controlled group (5.85 ± 2.65 vs. 4.55 ± 2.10 years, *P* < 0.001). In addition, the uncontrolled group had a significantly higher prevalence of allergic rhinitis (75.0% vs. 57.8%, *P* = 0.009), food allergy (22.1% vs. 11.7%, *P* = 0.037), and family history of asthma or allergic diseases (51.9% vs. 35.2%, *P* = 0.015). No statistically significant differences were observed in age at onset or eczema history between the two groups (both *P* > 0.05).

### 3.2 Treatment status, laboratory parameters, and airway characteristics

As shown in Table 2, significant differences were observed between the two groups in treatment patterns and recent exacerbation history. Children in the uncontrolled group were more likely to receive high-dose ICS therapy than those in the controlled group (54.8% vs. 18.0%, *P* < 0.001), and poor treatment adherence was also more common in the uncontrolled group (38.5% vs. 14.0%, *P* < 0.001). Moreover, the number of exacerbations during the previous year was significantly higher in the uncontrolled group than in the controlled group [1.00 (0.00, 2.00) vs. 0.00 (0.00, 1.00), *P* < 0.001].

**Table 2:** Treatment, laboratory, and airway characteristics.

Variables	Controlled (*n* = 128)	Uncontrolled (*n* = 104)	*P*-value
Treatment & exacerbation			
ICS dose (low/medium, high), *n* (%)	105/23 (82.0/18.0)	47/57 (45.2/54.8)	<0.001
Poor adherence, *n* (%)	18 (14.0%)	40 (38.5%)	<0.001
Exacerbations in past year	0.00 (0.00, 1.00)	1.00 (0.00, 2.00)	<0.001
Laboratory parameters			
Neutrophils (×10⁹/L)	3.05 ± 0.75	4.68 ± 1.17	<0.001
Lymphocytes (×10⁹/L)	2.81 ± 0.55	2.79 ± 0.72	0.484
Platelets (×10⁹/L)	279.66 ± 54.85	306.64 ± 67.83	0.001
Eosinophils (×10⁹/L)	0.23 (0.18, 0.29)	0.51 (0.40, 0.75)	<0.001
PIV	124.2 (94.4, 164.9)	383.1 (278.4, 551.0)	<0.001
SII	300.0 (232.4, 393.3)	648.3 (497.6, 925.7)	<0.001
NLR	1.11 (0.90, 1.30)	2.09 (1.63, 2.79)	<0.001
LDH (U/L)	195.3 ± 30.8	267.1 ± 44.0	<0.001
Vitamin D (ng/mL)	30.7 ± 5.5	20.4 ± 5.1	<0.001
Total IgE (IU/mL)	140.0 (87.5, 235.4)	328.1 (184.6, 553.6)	<0.001
CRP (mg/L)	1.29 (0.91, 1.66)	2.47 (1.59, 3.86)	0.011
Airway inflammation and lung function			
FeNO (ppb)	16.1 (12.5, 20.4)	39.9 (27.5, 54.9)	<0.001
FEV1%pred	95.9 ± 7.0	84.3 ± 12.9	<0.001
FEV1/FVC	84.5 ± 5.0	79.2 ± 6.3	<0.001
MMEF %pred	80.0 ± 15.3	66.3 ± 16.5	<0.001
FEF50% %pred	82.1 ± 13.5	67.5 ± 18.7	<0.001
BDR positive, *n* (%)	14 (10.9%)	29 (27.9%)	0.017

For laboratory findings, the uncontrolled group exhibited significantly higher neutrophil counts, platelet counts, eosinophil counts, and inflammatory composite indices than the controlled group. In particular, PIV was markedly elevated in the uncontrolled group [383.1 (278.4, 551.0) vs. 124.2 (94.4, 164.9), *P* < 0.001]. Similarly, SII and NLR were also significantly higher in the uncontrolled group (both *P* < 0.001), whereas lymphocyte counts did not differ significantly between groups (*P* = 0.484). In addition, serum LDH levels were significantly increased in the uncontrolled group (267.1 ± 44.0 vs. 195.3 ± 30.8 U/L, *P* < 0.001), while Vitamin D levels were significantly lower (20.4 ± 5.1 vs. 30.7 ± 5.5 ng/mL, *P* < 0.001). Total IgE and CRP levels were also significantly higher in the uncontrolled group than in the controlled group (both *P* < 0.05).

Regarding airway inflammation and pulmonary function, children with uncontrolled asthma had substantially higher FeNO levels than those with controlled asthma [39.9 (27.5, 54.9) vs. 16.1 (12.5, 20.4) ppb, *P* < 0.001]. In parallel, pulmonary function indices were significantly impaired in the uncontrolled group, including lower FEV1% predicted, lower FEV1/FVC ratio, lower MMEF % predicted, and lower FEF50% % predicted (all *P* < 0.001). Furthermore, the proportion of positive bronchodilator response (BDR) was significantly higher in the uncontrolled group than in the controlled group (27.9% vs. 10.9%, *P* = 0.017).

Taken together, these findings indicate that children with poor asthma control exhibited a more pronounced systemic inflammatory burden, greater metabolic disturbance, higher airway eosinophilic inflammation, and worse airway functional impairment.

### 3.3 Variable selection using LASSO regression

To identify the most relevant predictors and minimize overfitting, LASSO regression analysis was performed on 15 candidate variables covering multiple domains, including demographic and environmental factors (asthma duration, secondhand smoke exposure, allergic rhinitis, food allergy, and family history), treatment-related variables (ICS dose and poor adherence), laboratory parameters (PIV, LDH, Vitamin D, total IgE, and CRP), as well as airway inflammation and pulmonary function indices (FeNO, FEV1% predicted, and MMEF% predicted). To avoid potential multicollinearity, composite inflammatory indices (such as SII and NLR) and their individual components (neutrophils, lymphocytes, monocytes, and platelets) were not simultaneously included in the model. Given that PIV integrates these cellular components and provides a more comprehensive representation of systemic inflammation, it was retained as the primary inflammatory indicator in the analysis.

**Figure 1:**
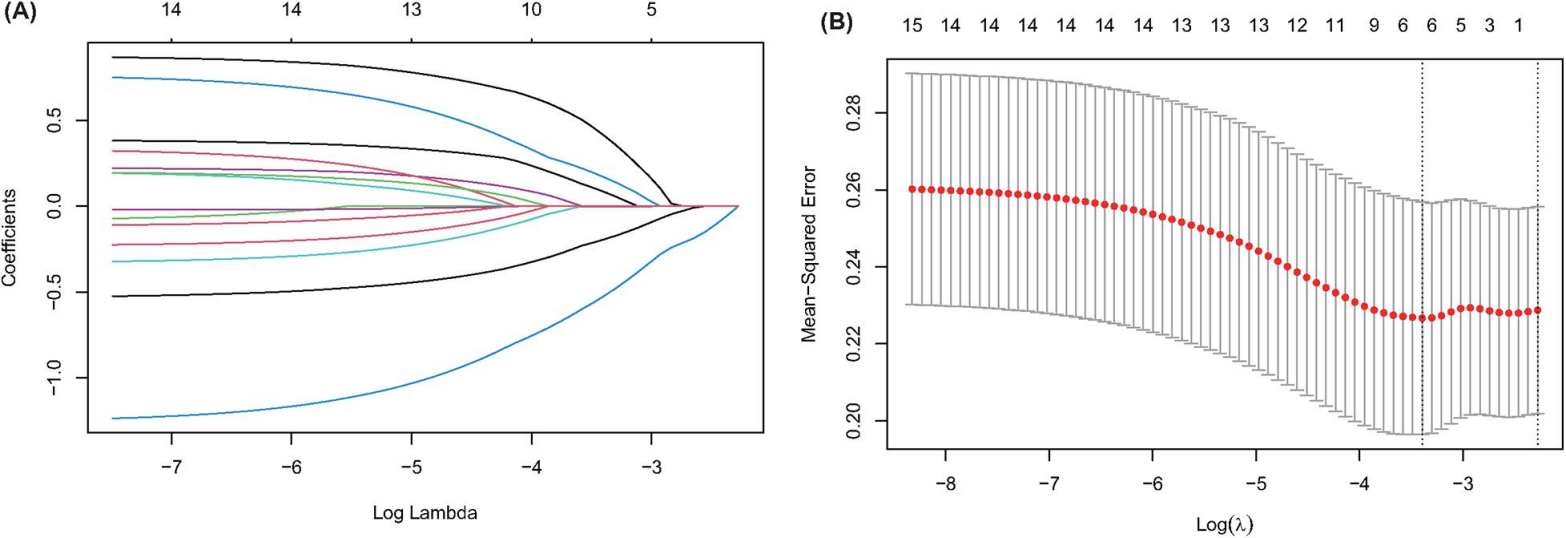
Variable selection using the least absolute shrinkage and selection operator (LASSO) regression model. **(A)** LASSO coefficient profiles of the 15 candidate variables. **(B)** Identification of the optimal penalty parameter via 10-fold cross-validation.

Using 10-fold cross-validation, the optimal penalty parameter (λ) was determined (Figure 1). At the selected λ value, six variables with non-zero coefficients were identified as the most informative predictors: PIV, LDH, FeNO, Vitamin D, asthma duration, and FEV1% predicted. These variables were subsequently included in the multivariate logistic regression analysis.

### 3.4 Multivariate logistic regression analysis for uncontrolled asthma

Multivariate logistic regression analysis was performed to determine independent risk factors for uncontrolled asthma (Table 3). The results showed that PIV (OR=1.008, 95% CI: 1.003–1.013, *P* < 0.001), LDH (OR=1.043, 95% CI: 1.025–1.061, *P* < 0.001), and FeNO (OR=1.056, 95% CI: 1.031–1.082, *P* < 0.001) were independently associated with an increased risk of poor asthma control.

**Table 3:** Multivariate logistic regression for predicting uncontrolled asthma.

Variables	Coefficient (*β*)	OR (95% CI)	*P*-value
PIV	0.008	1.008 (1.003–1.013)	<0.001
LDH	0.042	1.043 (1.025–1.061)	<0.001
FeNO	0.055	1.056 (1.031–1.082)	<0.001
Vitamin D	−0.115	0.891 (0.835–0.951)	0.001
Asthma Duration	0.224	1.251 (1.045–1.498)	0.015
FEV1% pred	−0.048	0.953 (0.912–0.996)	0.032

In contrast, higher Vitamin D levels (OR=0.891, 95% CI: 0.835–0.951, *P* = 0.001) and higher FEV1% predicted (OR=0.953, 95% CI: 0.912–0.996, *P* = 0.032) were identified as protective factors against uncontrolled asthma. Additionally, longer asthma duration was significantly associated with an increased risk of poor asthma control (OR=1.251, 95% CI: 1.045–1.498, *P* = 0.015).

These findings suggest that systemic inflammation (PIV), metabolic disturbance (LDH), airway inflammation (FeNO), and disease chronicity collectively contribute to poor asthma control, whereas better lung function and adequate Vitamin D levels may exert protective effects.

### 3.5 Predictive performance of biomarkers and combined model

Receiver operating characteristic (ROC) curve analysis was performed to evaluate the predictive performance of individual biomarkers and the combined model for identifying uncontrolled asthma (Table 4).

**Table 4:** Receiver operating characteristic (ROC) analysis of individual biomarkers and the combined model for predicting uncontrolled asthma.

Predictor(s)	AUC (95% CI)	Cut-off	Sensitivity (%)	Specificity (%)	Youden Index	P^a^
Individual Indicators						
PIV	0.781 (0.705–0.853)	265.45	68.8	76.7	0.455	<0.001
LDH (U/L)	0.755 (0.682–0.828)	232.5	72.1	70.3	0.424	<0.001
FeNO (ppb)	0.764 (0.685–0.843)	28.5	75	71.9	0.469	<0.001
Clinical Baseline						
Baseline Model^b^	0.732 (0.658–0.806)	—	65.4	72.3	0.377	<0.001
Combined Model						
Total Model^c^^,^ ^d^	0.886 (0.835–0.937)	0.485	85.6	86.2	0.718	<0.001

a P-value indicates the statistical significance of the Area Under the Curve (AUC) compared with the reference line (AUC = 0.5).

b Baseline Model: includes only asthma duration and FEV1% predicted.

c Total Model: includes PIV, LDH, FeNO, Vitamin D, asthma duration, and FEV1% predicted.

d P-value derived from the DeLong test indicated that the AUC of the Total Model was significantly higher than both individual FeNO (*P* < 0.001) and the Baseline Model (*P* < 0.001).

Among the individual indicators, PIV demonstrated an AUC of 0.781 (95% CI: 0.705–0.853), with a sensitivity of 68.8% and specificity of 76.7% at the optimal cut-off value of 265.45. LDH showed an AUC of 0.755 (95% CI: 0.682–0.828), with a sensitivity of 72.1% and specificity of 70.3% at a cut-off of 232.5 U/L. FeNO yielded an AUC of 0.764 (95% CI: 0.685–0.843), with a sensitivity of 75.0% and specificity of 71.9% at a cut-off value of 28.5 ppb.

The baseline model, including only asthma duration and FEV1% predicted, achieved a moderate predictive performance with an AUC of 0.732 (95% CI: 0.658–0.806). Notably, the combined model incorporating PIV, LDH, FeNO, Vitamin D, asthma duration, and FEV1% predicted demonstrated the highest predictive accuracy, with an AUC of 0.886 (95% CI: 0.835–0.937), a sensitivity of 85.6%, and a specificity of 86.2%. Formal comparisons using the DeLong test confirmed that the AUC of the combined model was significantly superior to that of FeNO alone (difference in AUC = 0.122, *P* < 0.001) and the clinical baseline model (difference in AUC = 0.154, *P* < 0.001).

These results indicate that the combined model significantly outperformed individual biomarkers and the baseline clinical model, highlighting the added value of integrating systemic inflammatory, metabolic, and airway-specific markers for predicting poor asthma control.

### 3.6 Construction and validation of the nomogram model

**Figure 2:**
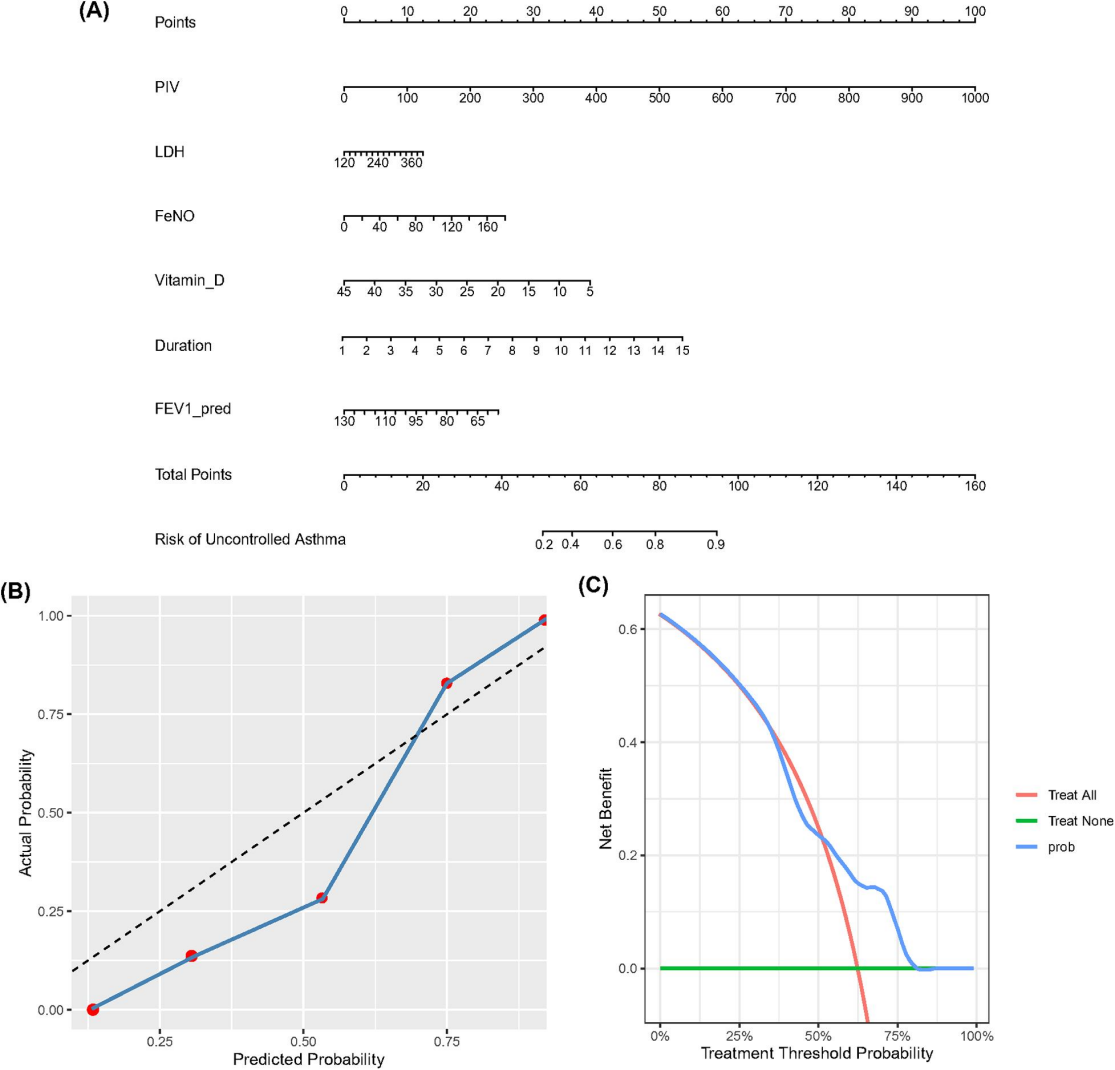
Construction and validation of the multidimensional nomogram for predicting uncontrolled asthma in school-aged children. **(A)** A predictive nomogram integrating systemic, metabolic, and airway-specific indicators. **(B)** Calibration curve demonstrating the agreement between the predicted probability and actual outcomes. **(C)** Decision curve analysis (DCA) evaluating the clinical utility of the nomogram. The *Y*-axis represents the net benefit.

A nomogram integrating PIV, LDH, FeNO, Vitamin D, asthma duration, and FEV1% predicted was constructed based on the multivariate logistic regression model to provide individualized risk estimation for uncontrolled asthma (Figure 2A). In the nomogram, each predictor was assigned a specific score proportional to its regression coefficient, and the total points corresponded to the predicted probability of poor asthma control. Among the included variables, asthma duration contributed the largest proportion to the total score, followed by FeNO and LDH, whereas PIV showed a relatively smaller contribution. Higher levels of PIV, LDH, FeNO, and longer asthma duration were associated with an increased risk of uncontrolled asthma, while higher Vitamin D levels and better lung function (FEV1% predicted) were associated with a decreased risk.

The calibration curve demonstrated good agreement between the predicted probabilities and the observed outcomes (Figure 2B). The apparent AUC was 0.886, and the internal bootstrap-corrected AUC was 0.869. The calibration validation demonstrated an optimal recalibration slope of 0.972 and an intercept of 0.018, indicating no significant overfitting. The calibration line was close to the ideal 45-degree reference line across different risk levels, indicating satisfactory calibration performance of the model.

Decision curve analysis (DCA) further evaluated the clinical utility of the nomogram (Figure 2C). The results showed that the nomogram provided a higher net benefit than both the “treat-all” and “treat-none” strategies across a wide clinical threshold probability range of 10% to 90%, suggesting that the model has favorable clinical applicability for real-time risk stratification and decision-making in children with asthma.

## 4 Discussion

In our present study, we developed and internally validated a multidimensional prediction model for poor asthma control in school-aged children by integrating systemic inflammatory, metabolic, airway-specific, and functional indicators. Several important findings emerged. First, children with uncontrolled asthma exhibited a more pronounced inflammatory and clinical burden, characterized by higher PIV, LDH, FeNO, and poorer lung function. Second, LASSO regression identified six key predictors-PIV, LDH, FeNO, Vitamin D, asthma duration, and FEV1% predicted-which remained independently associated with poor asthma control in multivariable analysis. Third, although PIV, LDH, and FeNO each showed moderate discriminative ability, the combined model substantially improved prediction performance, yielding an AUC of 0.886. Finally, the nomogram based on these predictors demonstrated favorable calibration and clinical net benefit, supporting its potential utility as a practical tool for individualized risk stratification in pediatric asthma. Collectively, these findings suggest that poor asthma control in school-aged children is better captured by an integrated “systemic inflammation-metabolic disturbance-airway inflammation-lung function” framework than by any single biomarker alone.

A key strength of this study is the inclusion of PIV as a composite marker of systemic immune-inflammatory activation. Asthma has traditionally been considered an airway-centered disease, yet growing evidence indicates that it also involves complex systemic immune dysregulation extending beyond localized bronchial inflammation (31–33). Neutrophils, monocytes, lymphocytes, and platelets all participate in different aspects of asthma pathobiology, including epithelial injury, cytokine amplification, leukocyte recruitment, and airway remodeling (34–36). Compared with single-cell indices such as NLR or SII, PIV theoretically provides a broader reflection of the balance between innate inflammatory activation and adaptive immune regulation. In this context, our finding that elevated PIV independently predicted poor asthma control is biologically plausible. Although PIV has been more widely studied in oncology and systemic inflammatory conditions, our results support the view that it may also serve as an accessible surrogate of inflammatory complexity in pediatric asthma. This interpretation is indirectly supported by previous pediatric studies showing that CBC-derived markers such as NLR and SII are elevated in children with asthma or asthma exacerbations (37, 38). Since PIV integrates several of these components into a single index, it may better reflect the multidimensional inflammatory milieu associated with uncontrolled disease.

Another notable finding is the independent contribution of LDH to poor asthma control. LDH is a cytoplasmic enzyme involved in the interconversion of pyruvate and lactate and is widely regarded as a marker of tissue injury, metabolic stress, and inflammatory activation (39, 40). In asthma, chronic inflammatory stimulation and repeated epithelial damage can promote metabolic reprogramming, cellular stress, and abnormal repair, thereby contributing to disease persistence and progression (41, 42). Prior studies have also suggested that LDH may reflect inflammatory activity in asthma, particularly during exacerbations (43, 44). Our observation that LDH remained an independent predictor after adjustment for other biomarkers indicates that metabolic disturbance may provide information beyond traditional inflammatory indices alone. This is clinically meaningful because asthma control is not determined solely by eosinophilic airway inflammation; it is also shaped by tissue injury, altered cellular energetics, and structural airway changes. Thus, the inclusion of LDH in the model broadens the pathophysiological scope of risk assessment and supports the idea that uncontrolled asthma involves both immunologic and metabolic dysregulation.

FeNO is one of the most established noninvasive biomarkers in asthma and represents an important airway-specific component of our model. Current guideline documents and expert statements recognize FeNO as a useful marker of type 2 eosinophilic inflammation, corticosteroid responsiveness, and, in selected contexts, future exacerbation risk (45–47). In our study, FeNO was markedly elevated in the uncontrolled group and independently associated with poor asthma control, which is consistent with its known relationship to persistent eosinophilic airway inflammation. However, FeNO alone achieved only moderate predictive accuracy. This is also in line with prior studies showing that FeNO is informative but not sufficient as a stand-alone biomarker, because its levels are influenced by asthma phenotype, atopy, allergen exposure, treatment status, and environmental context (48, 49). Therefore, the main value of FeNO in our study may lie not in replacing clinical evaluation, but in complementing systemic and functional markers within a multidimensional model. To rigorously justify this complementary approach and address the incremental value of our model over current standard tools, we explicitly quantified the predictive performance gaps. Compared to relying solely on FeNO (AUC = 0.764) or a standard clinical baseline involving lung function (AUC = 0.732), our combined multidimensional model achieved an absolute AUC increase of 0.122 and 0.154, respectively. This substantial gain in both sensitivity (increasing to 85.6%) and specificity (increasing to 86.2%) directly demonstrates that the integration of systemic inflammatory and metabolic markers offers significant incremental predictive power beyond conventional single-domain clinical tools.

Vitamin D emerged as a protective factor in the present study, with lower levels associated with poor asthma control. This finding accords with a substantial body of literature linking Vitamin D deficiency to enhanced airway inflammation, increased airway hyperresponsiveness, impaired lung function, and reduced responsiveness to corticosteroids (50, 51). Mechanistically, Vitamin D has been implicated in multiple immunoregulatory processes, including promotion of regulatory T-cell activity, suppression of exaggerated Th2/Th17 responses, modulation of epithelial barrier integrity, and attenuation of airway smooth muscle proliferation (52, 53). Although interventional evidence remains somewhat heterogeneous, several meta-analyses and reviews suggest that Vitamin D supplementation may reduce exacerbation risk in at least some subsets of patients with asthma, particularly among corticosteroid-treated populations or those with low baseline Vitamin D levels (54, 55). In this context, our results suggest that Vitamin D may contribute useful adjunctive information for identifying children at risk of poor disease control.

The clinical variables retained in the final model also deserve consideration. Longer asthma duration was positively associated with uncontrolled asthma, whereas higher FEV1% predicted was protective. These findings are consistent with the concept that chronicity and impaired lung function reflect cumulative disease burden and possibly ongoing airway remodeling (56, 57). Structural airway changes in childhood asthma-including epithelial disruption, basement membrane thickening, smooth muscle alterations, and abnormal repair-have been linked to poorer clinical outcomes, reduced treatment response, and persistent airflow limitation (58, 59). Likewise, previous cohort studies suggest that children with asthma are at risk for unfavorable lung function trajectories into adolescence and adulthood, especially when inflammatory burden remains high and disease control is suboptimal (60, 61). Our findings therefore reinforce the clinical importance of integrating both biological activity and physiological impairment when estimating the risk of poor asthma control.

One of the most clinically relevant observations in this study is that the combined model substantially outperformed the individual biomarkers and the baseline clinical model. This supports the notion that asthma control is a multidimensional construct that cannot be fully explained by any single biological pathway. PIV mainly reflects systemic inflammatory activation, LDH captures metabolic and tissue-injury signals, FeNO reflects airway eosinophilic inflammation, Vitamin D adds an immunomodulatory dimension, and FEV1% predicted and asthma duration reflect cumulative functional and clinical burden. Their integration yielded markedly improved discrimination and favorable calibration, suggesting that these variables capture complementary, rather than redundant, domains of disease biology. More broadly, our findings align with the growing interest in multivariable and machine-learning-assisted prediction approaches in pediatric asthma, which aim to move beyond conventional symptom-only assessment toward more precise risk profiling. From a translational perspective, the variables included in our model are all obtainable in routine clinical settings, which enhances the feasibility of implementation.

Recently, several pivotal studies have successfully leveraged machine learning pipelines to untangle the clinical heterogeneity of asthma. For instance, Zhao et al. successfully utilized a similar analytical framework—incorporating LASSO regression for feature selection followed by multivariate logistic regression—to develop a clinical nomogram predicting the long-term persistence of school-age asthma in preschool children, demonstrating stable moderate-to-good discrimination (62). While their prognostic model relied primarily on conventional clinical histories and atopic comorbidities, our study extends this robust statistical methodology to school-aged children by integrating objective, multi-dimensional laboratory biomarkers to evaluate contemporaneous disease control status. Furthermore, the capacity of machine learning to uncover hidden, multi-systemic associations in asthma has been highlighted by Huang et al., who applied an XGBoost model coupled with SHAP to a large national cohort (63). Their feature importance analysis revealed that systemic inflammatory parameters (such as peripheral eosinophil percentages and C-reactive protein) and nutritional/metabolic factors are heavily weighted in determining asthma outcomes. This robustly validates our clinical rationale for incorporating systemic immune-inflammation (PIV) and metabolic distress (LDH) indicators alongside airway-specific parameters (FeNO). From a translational perspective, the variables included in our model are all obtainable in routine clinical settings, which enhances the feasibility of implementation.

The nomogram developed in this study may have practical clinical value. Because all included variables are readily obtainable in routine practice and are noninvasive or minimally invasive, the model may help identify children at increased risk of poor asthma control during follow-up. These patients may benefit from closer monitoring, improved adherence management, reassessment of environmental exposures and comorbidities, and earlier treatment adjustment. Moreover, the DCA results indicate that the model provides favorable net clinical benefit across a range of threshold probabilities, supporting its potential role as a pragmatic tool for risk stratification and individualized management in pediatric asthma.

Several limitations should be acknowledged. First, this was a single-center retrospective study, which may introduce selection bias and limit generalizability. Second, the sample size, although adequate for exploratory model development, remains modest; therefore, the stability of the model should be further tested in larger and more diverse cohorts. Third, external validation was not performed, and the predictive performance observed in the present dataset may be somewhat optimistic. Fourth, some potentially relevant determinants of asthma control—such as allergen sensitization profile, environmental pollutant exposure, viral infection history, and detailed adherence quantification—were not fully incorporated. Additionally, while prominent baseline clinical confounders such as adherence, smoke exposure, and exacerbation history differed between groups, they were excluded by the automated LASSO process to achieve model parsimony and prevent multicollinearity; while this yields a streamlined, biomarker-centric tool, it represents a clinical limitation. Fifth, a conceptual limitation of this study is that because of its retrospective and contemporaneous nature, the model serves as an objective assessment tool for current asthma control status rather than a forward-looking predictor independent of baseline disease severity. Since lung function and FeNO are heavily linked to current control definitions, their high coefficients in the nomogram reflect this simultaneous clinical relationship. Finally, because asthma is heterogeneous and dynamic, longitudinal studies are needed to determine whether changes in PIV, LDH, FeNO, and Vitamin D over time can improve prediction of subsequent loss of control or exacerbation risk.

In summary, our study indicates that poor asthma control in school-aged children is associated with the interplay of systemic inflammation, metabolic stress, airway eosinophilic inflammation, disease chronicity, and functional impairment. By integrating PIV, LDH, FeNO, Vitamin D, asthma duration, and FEV1% predicted, we established a nomogram with good discrimination, calibration, and potential clinical utility. These findings not only expand the understanding of the multidimensional biology underlying uncontrolled pediatric asthma, but also provide a practical basis for early risk stratification and individualized management. Future multicenter prospective studies with external validation and longitudinal biomarker assessment are warranted to confirm and refine this model.

## Data Availability

The original contributions presented in the study are included in the article/Supplementary Material, further inquiries can be directed to the corresponding author.
